# Early Environmental and Biological Influences on Preschool Motor Skills: Implications for Early Childhood Care and Education

**DOI:** 10.3389/fpsyg.2021.725832

**Published:** 2021-08-13

**Authors:** Elena Escolano-Pérez, Carmen Rosa Sánchez-López, Maria Luisa Herrero-Nivela

**Affiliations:** ^1^Department of Psychology and Sociology, University of Zaragoza, Zaragoza, Spain; ^2^Department of Clinical Psychology, Psychobiology and Methodology, University of La Laguna, San Cristóbal de La Laguna, Spain

**Keywords:** motor skills, early childhood, delivery mode, feeding type, sex, relative age effect, observational methodology, early childhood care and education

## Abstract

Early motor skills underpin the more complex and specialized movements required for physical activity. Therefore, the design of interventions that enhance higher levels of early motor skills may encourage subsequent participation in physical activity. To do so, it is necessary to determine the influence of certain factors (some of which appear very early) on early motor skills. The objective of this study was to examine the influence of some very early environmental variables (delivery mode, feeding type during the first 4 months of life) and some biological variables (sex and age in months) on preschool motor skills, considered both globally and specifically. The sample was composed by 43 preschool students aged 5–6 years. The participant's parents completed an *ad hoc* questionnaire, reporting on delivery mode, feeding type, sex, and age in months. The children's motor skills were assessed using observational methodology in the school setting, while the children participated in their regular motor skills sessions. A Nomothetic/Punctual/Multidimensional observational design was used. Results revealed that certain preschool motor skills were specifically influenced by delivery mode, feeding type, sex, and age. Children born by vaginal delivery showed higher scores than children born via C-section in throwing (*p* = 0.000; *d* = 0.63); total control of objects (*p* = 0.004; *d* = 0.97); total gross motor skills (*p* = 0.005; *d* = 0.95); and total motor skills (*p* = 0.002; *d* = 1.04). Children who were exclusively breastfed outperformed those who were formula-fed in throwing (*p* = 0.016; *d* = 0.75); visual-motor integration (*p* = 0.005; *d* = 0.94); total control of objects (*p* = 0.002; *d* = 1.02); total gross motor skills (*p* = 0.023; *d* = 0.82); and total motor skills (*p* = 0.042; *d* = 0.74). Boys outperformed girls in throwing (*p* = 0.041; *d* = 0.74) and total control of objects (*p* = 0.024; *d* = 0.63); while the opposite occurred in static balance (*p* = 0.000; *d* = 1.2); visual-motor coordination (*p* = 0.020; *d* = 0.79); and total fine motor skills (*p* = 0.032; *d* = 0.72). Older children (aged 69–74 months) obtained higher scores than younger ones (aged 63–68 months) in dynamic balance (*p* = 0.030; *d* = 0.66); visual-motor integration (*p* = 0.034; *d* = 0.63); and total balance (*p* = 0.013; *d* = 0.75). Implications for early childhood care and education are discussed since this is a critical period for motor skill development and learning.

## Introduction

The World Health Organization has declared that 81% of all school-aged children fail to engage in the minimum recommended amount of daily physical activity (World Health Organization, 2010; Bull et al., 2020). This means that a large number of children do not receive the many physical, mental, and socio-emotional benefits of regular physical activity. This can be corrected, however, since physical activity (or the lack thereof) is a modifiable behavior. An initial step is to identify and determine the factors underlying this lack of physical activity in children. These variables include the level of motor skills acquired during early childhood (De Niet et al., 2021; Moghaddaszadeh and Belcastro, 2021). Motor skills include the movement and coordination of one's muscles and body (Matheis and Estabillo, 2018). They are classified into two groups: (1) Gross motor skills and (2) Fine motor skills (Gonzalez et al., 2019; Goodway et al., 2019; Meylia et al., 2020). (1) Gross motor skills refer to developmental aspects associated with the child's ability to move using their large muscle groups to perform activities such as walking and jumping. (2) Fine motor skills refer to precise movements using smaller muscle groups to perform more delicate tasks such as picking up small objects, threading beads, and writing. They require control and coordination of the distal musculature of the hands and fingers. Both gross and fine motor skills can be divided into more specific typologies (Goodway et al., 2019; Bolger et al., 2020; Meylia et al., 2020). (1) Three types of gross motor skills have been established: (1.1) Locomotor skills: these are movements having the fundamental objective of moving the body from one point in space to another, such as: running, jumping, rolling, etc. (1.2) Balance: this is the ability to maintain a controlled position or posture during a specific task. Here, differentiation is made between: (1.2.1) Static balance: it is the ability to maintain postural stability and orientation with the center of mass over the base of support while the body is at rest. It is necessary, for example, to perform squats; and (1.2.2) Dynamic balance: it refers to the same ability to maintain postural stability and orientation with the center of mass over the base of support but while the body parts are in motion. An example of dynamic balance task is stair climbing. (1.3) Object control skills: skills that allow the individual to move or receive objects, be it with the feet, hands, or even the body. Differentiation is made between: (1.3.1) Propulsive skills: they involve sending an object away from the body, such as throwing, or batting a ball; and (1.3.2) Receptive skills: they involve receiving an object, such as catching a ball, or a frisbee (Kokstejn et al., 2017; Bolger et al., 2020). As for (2) Fine motor skills, two separate elements have been established: (2.1) Visual-motor coordination (also referred to as Fine motor coordination): it refers to small muscle movements with a visual component. It includes abilities such as finger dexterity, motor sequencing, and fine motor speed and accuracy. These skills are used in tasks such as building with blocks; finger tapping, and imitative hand movements. (2.2) Visual-motor integration (also called Visual-spatial integration or Fine motor integration): it involves the organization of small muscle movements in the hand and fingers with the processing of visual stimuli. It implies that visual information from the environment is processed and integrated using fine motor movements. It requires more visual perception than Visual-motor coordination. Visual-motor integration skills are often captured by tasks that involve writing and copying (Goodway et al., 2019).

Early motor skills are essential for subsequent physical activity. They are the basis of more advanced, complex, and specialized movements needed to participate in games, sports, and other context-specific physical activity (Chang et al., 2020; Moghaddaszadeh and Belcastro, 2021). Therefore, promoting and obtaining a suitable level of early motor skills is a positive element that may stimulate and enhance the onset and maintenance of physical activity. Children with good motor skills are perceived as being competent, leading to increased enjoyment and engagement in more and wider variety of motor and physical activity experiences. Increased physical activity provides more opportunities to promote motor skill development. Therefore, a positive spiral or dynamic relationship is evident between motor skills and physical activity (Stodden et al., 2008). On the other hand, less-skilled children will have a lower perceived competence and will perceive many tasks as being more difficult and challenging, therefore being less likely to engage in them. Hence, having good motor skills, even in early childhood, may contribute to becoming a physically active individual, or even an elite athlete (De Niet et al., 2021).

Despite the clear importance of these early motor skills in the life and development of children, they tend to be overlooked on a research and practical/educational level (Lopes et al., 2021). This has led to an increase in the number of children with poor motor skills and an upward trend of motor difficulties over recent years (Honrubia-Montesinos et al., 2021; Lopes et al., 2021).

One of the issues that seems to have contributed to this lack of research and promotion of early childhood motor skills is the misconception that they will develop naturally over time. However, to attain an appropriate motor skill level, these skills must be learned, practiced, and reinforced over time (Honrubia-Montesinos et al., 2021; Moghaddaszadeh and Belcastro, 2021). Preschool years are an especially important life phase for the development and learning of motor skills (Wang et al., 2020; Lopes et al., 2021). During these years, development occurs quickly and it is closely linked to the quality and quantity of the stimuli received by the children. Therefore, during early childhood children should be offered enriched environments, allowing them to achieve their full motor potential (Lopes et al., 2021; Moghaddaszadeh and Belcastro, 2021). Early Childhood Education classrooms are an ideal context for this since a large number of children attend these schools, spending many hours there (European Commission/EACEA/Eurydice, 2019; Spanish Ministry of Educational Professional Training, 2020).

However, we should note that, for Early Childhood Education experiences to be effective, they should be intentionally designed taking into account the child's current level of development (Darling-Hammond et al., 2020). However, a problem arises for educators. Even children in the same academic year and enrolled in the same class may display different motor skills levels, given their distinct characteristics and past and present experiences. Numerous and diverse factors may affect motor skill levels in children (Wang et al., 2020). Learning more about these potential factors and their influences on children's motor skills is necessary to design individualized interventions that optimize motor skills for all children.

Given all of this, as well as the current literature on the topic, the purpose of this study was to provide knowledge as to the influence of certain variables (some of which are present from a very early age) on global and specific motor skills, on preschool-age children (5- and 6-year-olds). The following variables of influence on preschool motor skills were considered: delivery mode, feeding type during the four first months of life, sex, and age (specifically, the relative age effect), since, according to the literature, there is still an ongoing discussion regarding its relationship with motor skills.

Each of these variables is discussed below.

### Delivery Mode

A possible impact of delivery mode on the neurodevelopment of children has been considered. The mode of delivery has been directly related to biochemical and structural changes in the central nervous system, although their consequences are not well-known. Thus far, literature on this area (especially referring to the influence of the delivery mode on motor skills) has been inconclusive, since studies are scarce and they offer conflicting results (Blazkova et al., 2020; Takács et al., 2020).

Vaginal delivery is considered to be the ideal mode of delivery for the child's development. It is the most natural delivery mode and tends to lead to a lower number of complications for both mother and child (World Health Organization, 2018). In this mode of delivery, certain inherent mechanisms may be produced, possibly triggering certain protective and strengthening processes for the child's appropriate development (Tribe et al., 2018). Over recent years, however, the rate of cesarean deliveries (C-sections) has increased considerably in numerous countries (World Health Organization, 2018). It has been due an overuse of the procedure, and not to medical indications (World Health Organization, 2018), such as mothers' wishes to have a planned birth (King, 2021). Many studies have warned of the harmful consequences that this may entail, since, like any other surgery, C-sections are associated with short- and long-term risks that may persist years after the intervention and which may affect the child's health and development (Chojnacki et al., 2019; King, 2021). Specifically, about the effect of C-sections on motor development, further research is necessary given the literature is limited and does not offer conclusive results.

Some studies have found no evidence to affirm that children born by C-section display poorer gross and fine motor skills than those born by vaginal delivery (Zhou et al., 2019; Takács et al., 2020).

Other studies have found the opposite results, suggesting that delivery mode affects the child's motor development. Rebelo et al. (2020) studied the influence of delivery mode on motor skills (both gross and fine) in children aged 12–48 months. Their results indicated that: (1) children born via vaginal delivery had better motor skills, both gross and fine, as compared to those born via C-section. More specifically, in older children (36–48 months), differences based on delivery mode were statistically significant in object control, visual-motor coordination, and visual-integration skills, as well as in the score on total gross motor skills and total fine motor skills. No statistically significant differences were found for locomotor and balance skills; (2) the effect of delivery mode on motor skills became more pronounced as the children became older. Blazkova et al. (2020) also found that the mode of delivery had a major effect on visual-integration skills in 5-year-olds: those born via vaginal delivery had higher visual-motor integration than those born by C-section. No additional measures regarding motor skills were included in said study.

In summary, along with the disparate results found between studies regarding the influence (or lack of) of delivery mode on motor skills, it has been found that most of the studies offer only partial results and fail to consider all specific gross and fine motor skills that have been identified in the literature (and explained above). Given these limitations and lack of knowledge, it was decided to include this variable in this study.

### Feeding Type

Appropriate feeding practices are vital for children's optimal growth and development. Breastfeeding is recognized as the gold standard for infant nutrition (Chen et al., 2021). Many of the components of breast milk offer multiple benefits to the child's health, growth, and development, over the middle- and long-term. Breastfeeding has been associated with appropriate cerebral development, improved immunity, and a decreased risk of infections, metabolic diseases (including obesity and diabetes), asthma, and cardiovascular risk. It also may result in better mental health, improved cognitive and language development and academic performance (Grace et al., 2017; Jardí et al., 2018). Few studies exist, however, regarding its effect on motor development (Hernández Luengo et al., 2019). And said studies have focused more on analyzing the effects of a longer or shorter duration of breastfeeding on motor development rather than on the effects of breastfeeding as compared to other types of infant feeding (such as formula feeding). Among the limited studies that have considered this topic, results have been non-conclusive. Moreover, studies analyzing the effects beyond 3 years are even more few.

Bellando et al. (2020) found that, at the age of 3 months, breastfed infants displayed better motor development than formula-fed infants. However, at 12 and 24 months, no differences were found between both groups. Similar results have been found by Michels et al. (2017), who suggested that the type of feeding during the first 4 months of life does not impact the ages at which gross motor milestones (standing and walking alone) are achieved.

Other studies have offered distinct results, suggesting that associations exist between infant feeding type and motor skills. Jardí et al. (2018) found that children who were exclusively breastfed for the first 4 months of life (as compared to those who were formula-fed or mixed-fed during that time) displayed better motor development at 6 months and 1 year of age. Results found by Kádár et al. (2021) suggest the same. At 1 year of age, children who were exclusively breastfed for 6 months showed the lowest incidence of delays in their motor development. Those who were exclusively formula-fed during this time had the highest incidence of delays.

This set of studies presents some important results, although motor development is only considered in a general manner, and without differentiating between different motor skills. Very few studies have made this differentiation and those that have revealed discrepancies as to whether or not the infant feeding type influences gross and fine motor skills. Sacker et al. (2006) reported that at 9 months of age, children who had never been breastfed were the most likely to have delays in motor development (both gross and fine). Similarly, and further supporting the positive effects of breastfeeding as compared to other infant feeding types for gross and fine motor skills, Dee et al. (2007) found that breastfeeding was a protective factor against developmental delays (for both gross and fine motor skills) in children aged 1–5. The results of Leventakou et al. (2015) were somewhat different, however. They found that at 18 months, no differences existed in the gross motor skill level of children who were never breastfed as compared to those who were. On the other hand, differences were found between children in terms of fine motor skills, which were lower in children who were never breastfed. Therefore, according to these authors, fine motor skills are more sensitive to the effects of feeding than gross motor skills. We are unaware of studies that have analyzed the effects of infant feeding type on different specific gross and fine motor skills.

Given the wide variety of results and this gap, there is clearly a need for additional research to determine the impact of early feeding type on motor skills, specifically considering its influence on different specific gross and fine motor skills. Existing studies have failed to consider this issue.

### Sex

Numerous studies have suggested differences in the motor skills of boys and girls (Kokstejn et al., 2017; Matarma et al., 2020; Mecías-Calvo et al., 2021). These differences have been primarily explained by the different stereotyped activities, sporting or other, that are carried out by the different sexes, and not by differences in their physical characteristics (body type, body composition, strength, and limb length), since, before puberty, these characteristics are quite similar in both boys and girls (Bolger et al., 2020; Matarma et al., 2020). Some studies, however, have failed to find differences in preschool motor skills between boys and girls (Martínez-Moreno et al., 2020).

Discrepancies exist even among those who defend the idea that there are differences in motor skills according to sex. The influence of sex on infant motor skills appears to depend on the specific motor skill at hand, but there is no consensus as to the specific associations. Thus, discrepancies exist as to which sex displays better performance on each of the motor skills.

Regarding gross motor skills, some studies have found that boys outperform girls (Bolger et al., 2018, 2020; Wang et al., 2020), while other studies have suggested that girls outperform boys (Matarma et al., 2020) and others have found no differences between both sexes (Peyre et al., 2019; Martínez-Moreno et al., 2020). In terms of fine motor skills, girls have been found to have better performance than boys (Kokstejn et al., 2017; Peyre et al., 2019; Mecías-Calvo et al., 2021), although other studies have suggested that fine motor skills are very similar between both sexes (Martínez-Moreno et al., 2020).

These discrepancies regarding which motor skills present differences and which do not, and whether said differences favor boys or girls, become even greater when we consider the different specific skills making up the gross motor skills. Some studies have suggested that locomotor skills are higher in girls (Bolger et al., 2018, 2020; Wang et al., 2020), while other works claim that they are higher in boys (Robinson, 2010); and other studies have failed to detect any significant differences between both sexes (Bakhtiar, 2014; Foulkes et al., 2015; Barnett et al., 2016; Bolger et al., 2018, 2020). As for balance skills, some studies have shown that these skills are higher in girls (Venetsanou and Kambas, 2016; Kokstejn et al., 2017; Mecías-Calvo et al., 2021) while others indicate that they are similar for both sexes (Singh et al., 2015; Barnett et al., 2016). As for control object skills, some studies show higher levels in boys (Foulkes et al., 2015; Barnett et al., 2016; Venetsanou and Kambas, 2016; Kokstejn et al., 2017; Bolger et al., 2018, 2020; Mecías-Calvo et al., 2021) while others find similar levels between both sexes (LeGear et al., 2012; Bakhtiar, 2014). We are unaware of studies that have focused on the analysis of potential differences based on sex for the distinct specific preschool fine motor skills.

Given the disparity of results and this gap, additional research is clearly necessary in this area. Therefore, in our study, we have included the sex variable to analyze its influence on (global and specific) motor skills.

### Age

It is well-known that as children grow, their motor skills improve (Bolger et al., 2018). What is not so well-known is whether significant differences exist between the motor skills of children born in the same year. In Spain (where this research was conducted), the educational policy groups together children based on their date of birth, with all children born in the same natural year (January 1 to December 31) being grouped in the same academic year. This is an attempt to seek the minimum number of differences between children in the same academic year, and to offer appropriate experiences for all. However, in fact, this means of grouping leads to cases of an almost full year's difference in the age of some students who are in the same academic year (12 months minus 1 day). That is, there is a chronological age difference between children of the same cohort. The results of this phenomenon are referred to as the “relative age effect” (RAE) (Gladwell, 2008). The RAE refers to the effects of being relatively younger or older than peers. It may result in children who are born earlier in their year of birth outperforming children of the same cohort who were born later in the year. Therefore, being born later potentially puts these children at a disadvantage as compared to their peers with earlier birthdays. The size of the RAE is inversely correlated with age, such that the RAE is more prominent in early grades (Aune et al., 2018).

Some studies have found that even as early as in Early Childhood Education, children born at the beginning of the year displayed higher levels of gross and fine motor skills than their peers with later birthdays (Martínez-Moreno et al., 2020; Mecías-Calvo et al., 2021; Navarro-Patón et al., 2021). This appears to be due not only to their nervous and muscular system having matured for a longer period of time, but also to their increased opportunities for motor practices, experiences, and feedback; issues that may help to refine their motor skills (Bolger et al., 2020; Cupeiro et al., 2020).

Although these studies have revealed the existence of an RAE on preschool motor skills, it should be noted that their results also suggest that the RAE does not affect all of the preschool motor skills, with discrepancies arising when attempting to determine which motor skills have an RAE and which do not. In addition, the use of distinct assessment instruments and the consideration of different motor skills prevent the comparison of studies. Therefore, for example, Imbernón-Giménez et al. (2020) found an RAE on the control of objects, visual-motor integration, and total gross motor skill score. However, they did not find it on locomotor, balance, or total score for fine motor skills. Visual-motor coordination and the total motor skill score were not considered in this study. Mecías-Calvo et al. (2021) did not find results that coincide with those of prior authors, since they found an RAE on balance but not on object control. Other results of Mecías-Calvo et al. (2021)–referring to motor skills not considered by Imbernón-Giménez et al. (2020)–found the existence of an RAE on visual-motor coordination and total motor skills score. Navarro-Patón et al. (2021) found an RAE on object control –coinciding with Imbernón-Giménez et al. (2020)–, as well as for visual-motor coordination –coinciding with Mecías-Calvo et al. (2021)–. However, they did not find an RAE on balance –unlike Mecías-Calvo et al. (2021), but like Imbernón-Giménez et al. (2020)–. They also failed to find an RAE on the total motor skills score –an aspect that also diverges from Mecías-Calvo et al. (2021)–. More specific results were found by Imamoglu and Ziyagil (2017). These authors analyzed the RAE on locomotor and object control skills, detecting that only some, –not all– locomotion skills were affected by this effect. The object control skills, as suggested by Mecías-Calvo et al. (2021) did not reveal an RAE, unlike the results of Imbernón-Giménez et al. (2020) and Mecías-Calvo et al. (2021).

Given the wide variety of results, based on partial studies that do not consider all of the specific gross and fine motor skills identified in the literature, further research is necessary to determine which of the specific preschoolers' gross and fine motor skills are influenced by an RAE.

## Aim

The objective of this study was to analyze whether there were influences of delivery mode, type of feeding during the first 4 months of life, sex, and age (more precisely, the RAE) on motor skills (considered at both a global and specific level), assessed in 5- and 6-year-old preschoolers.

Based on the existing literature on this area, we proposed the following hypotheses:

- H_1_: Differences will be found in childhood motor skills based on the delivery mode: children born by vaginal delivery will have higher motor skills than children born by C-section.
- H_2_: Differences will be found in childhood motor skills based on the type of feeding during the first 4 months of life: children who were exclusively breastfed during this time will have higher motor skills than children fed with formulas or mixed-fed.
- H_3_: Differences will be found in childhood motor skills based on sex: boys will outperform girls on certain motor skills while, in other motor skills, the opposite will occur. Moreover, in other skills, no differences will be found between both sexes.
- H_4_: There will be an RAE on certain preschool motor skills: children born over the first half of the year will outperform their classmates who were born over the second half of the same year on some motor skills.

We believe that determining whether these variables have an influence on the motor skills of 5- and 6-year-olds may be of great assistance to educators as well as health, sports, and physical activity professionals, who may subsequently design more effective personalized interventions. These results may be relevant for policymakers when implementing public health, social, and educational policies that promote appropriate motor skill development from very early ages, thereby enhancing physical activity and healthy lifestyles in the children.

## Materials and Methods

### Methodology and Design

Data for this study are a subset of a broader research project focusing on the analysis of diverse childhood skills and competencies.

A multimethod and mixed methods approach was used (Elliott, 2007; Sánchez-Algarra and Anguera, 2013; Anguera et al., 2018a). The multimethod approach consisted of selective methodology to determine the delivery mode, feeding type during the first 4 months of life, sex, and age (a questionnaire was used for this), as well as information referring to the sample's inclusion/exclusion criteria (questionnaires and standardized batteries were used); and observational methodology to observe preschool motor skills in the school context while the children participated in their regular motor skills sessions. Our study was also carried out from a mixed methods approach because observational methodology itself is considered a mixed methods, since it integrates qualitative and quantitative elements in a succession of QUAL-QUAN-QUAL macro-stages (Sánchez-Algarra and Anguera, 2013; Anguera et al., 2018a, 2020a,b). In the first QUAL stage, an *ad hoc* observation instrument is built and applied to code the behaviors that are the subject of the study, taking into account the natural setting in which they occur. Then, in the QUAN stage, observational data quality is tested and analyses through quantitative techniques are carried out to respond to the study objectives. The quantitative results obtained are qualitatively interpreted in the third and last stage (QUAL stage), considering the research problem and the literature. All this permit a seamless integration.

Observational methodology plays an essential role in our study. It is a robust scientific method for analyzing regular behavior (like the motor behaviors studied in this work) in natural settings (such as the scholastic one, the context in which this research was carried out) (Suárez et al., 2018, 2020; Escolano-Pérez et al., 2019a,b; Anguera et al., 2020a,b; Sagastui et al., 2020). Furthermore, observational methodology is the most appropriate methodology for studying the behavior of young children (like those in this study who were 5 and 6 years of age) (Anguera, 2001; Early Head Start National Resource Center, 2013; Blanco-Villaseñor and Escolano-Pérez, 2017; Escolano-Pérez et al., 2017; Escolano-Pérez et al., 2019b).

Of the eight types of existing observational designs (Anguera et al., 2018b), we employed a Nomothetic/Punctual/Multidimensional design. It was: “Nomothetic” because various units of observation were studied (43 children); “Punctual” because for each child, an observation session was carried out to study each of the motor skills of interest in the study; and “Multidimensional” because different response levels were observed, that is, distinct aspects were observed regarding the gross and fine motor skills, thereby following the theoretical proposal of distinct authors (Matheis and Estabillo, 2018; Gonzalez et al., 2019; Goodway et al., 2019; Meylia et al., 2020). These response levels are reflected in the observation instrument used (available in the Supplementary Material).

The observation was active, based on scientific criteria, non-participatory and direct (the level of perceptibility of the behaviors was complete). It was performed by direct observation of recorded film (Anguera et al., 2018b).

### Participants

Preschool children aged 5 and 6 (N = 43: 15 boys and 28 girls; 34.88% and 65.12%, respectively) in the third year of Early Childhood Education (M_age_ = 68.6 months; SD_age_ = 3.59) from an intentionally selected public school participated in the study. The school was located in a middle-upper (socioeconomic) class neighborhood, in a city in the northeast of Spain.

All children had the following characteristics (meeting exclusion/inclusion criteria established for study participants): (1) absence of a history of pre, peri, or postnatal problems, neurological disease, sensory disturbance, mental or other clinically diagnosed impairment (such as attention-deficit hyperactivity disorder, developmental coordination disorder, developmental dysphasia, etc.) or special needs, according to the information provided by the parents of the children; (2) according to the school's management team, all participants were enrolled in the school since the 1st year of Early Childhood Education. That is, they were completing the entire second cycle of this educational stage (from 3 to 6 years of age) at this school; (3) they had appropriate IQ for their age, according to the assessment carried out by the research team using the BADyG-I (Battery of Differential and General Abilities I; Yuste and Yuste, 2001).

The study was part of a broader research project endorsed by the Research Unit of the University of Zaragoza. All participants were treated in accordance with the principles of the Declaration of Helsinki. Written informed consent was required from the children's parents.

### Instruments

#### Instruments Used for Selective Methodology

An *ad hoc* questionnaire to be completed by the participants' parents was used to determine the following: (1) Information related to the exclusion criterion referring to a history of pre, peri, or postnatal problems, neurological disease, sensory disturbance, mental or other clinically diagnosed impairment (such as attention-deficit hyperactivity disorder, developmental coordination disorder, developmental dysphasia, etc.), or special needs; (2) Information on the delivery mode, type of feeding during the first 4 months of life, sex, and age of each participant. More specifically, and regarding these variables, the questionnaire requested that the following be indicated: (a) delivery mode: select between vaginal delivery and C-section, according to the classification used in similar past studies, such as those by Khalaf et al. (2015) and Grace et al. (2017); (b) feeding type during the first 4 months of life: select between exclusive breastfeeding; exclusive formula or artificial milk feeding; and mixed-feeding (combination of breast and formula feeding), according to the classification proposed by other similar past studies (Tozzi et al., 2012; Michels et al., 2017; Jardí et al., 2018; Chojnacki et al., 2019). It should be noted that this age (4 months) was selected because, according to the studies conducted in Spanish contexts, this is a turning point in infant feeding. Most Spanish mothers tend to stop breastfeeding at this point, given that their maternity leave ends and they have to return to work. At this point, many mothers resort to other feeding options for their children (Jardí et al., 2018; Cabedo et al., 2019); (c) sex: select between masculine and feminine; (d) date of birth, indicating the day, month, and year. The questionnaire also contained a section for additional “considerations” allowing parents to clarify any responses.

To gather information referring to the inclusion criterion of being enrolled in the school since the 1st year of Early Childhood Education (that is, to be completing the entire second cycle of this educational stage in the school), another *ad hoc* questionnaire was used, to be completed by the preschool management team.

To determine whether all of the participants complied with the inclusion criterion of having an appropriate IQ for their age, the BADyG-I (Battery of Differential and General Abilities I; Yuste and Yuste, 2001) was used. It is one of the most widely used instruments in Spain (where the study was conducted) to measure student IQ, since it has suitable psychometric properties and provides a complete measurement including distinct verbal (Numerical-Quantitative Concepts, Information, and Graphic Vocabulary) and non-verbal (Reasoning with Figures, and Logic Puzzles) fields. BADyG-I offers a Verbal, Non-verbal, and General IQ.

#### Instruments Used for Observational Methodology

According to the GREOM (Guidelines for Reporting Evaluations Based on Observational Methodology; Portell et al., 2015), it is necessary to differentiate between recording (to record or code data) and observation (to observe a specific topic) instruments.

##### Recording instruments

The following recording instruments were used: (1) a video camera to record the children's motor sessions and (2) the free software Lince v.1.2.1 (Gabin et al., 2012) to code actions indicative of infant motor skills.

##### Observation instrument

We created a modified version of the original *ad hoc* observation instrument by Escolano-Pérez et al. (2020). The modifications included new categories, the elimination of other categories, and some more specific definitions. The observation instrument was a combination of a field format and systems of categories, given that the observational design was multidimensional (Anguera et al., 2018b). This observation instrument consists of a total of 26 criteria. Each criterion was broken down into a system of exhaustive, mutually exclusive categories. Overall, the observation instrument contained 82 categories. The selection of criteria and categories was based on the information provided by theoretical and empirical studies on childhood motor skills (Hestbaek et al., 2017; Oberer et al., 2017; Goodway et al., 2019; Haywood and Getchell, 2019); the Spanish Early Childhood Education curriculum, which determines the motor skills worked on during this educational stage (Education Science Ministry of Spanish Government, 2007), and the information obtained from the reality observed. The observation instrument is fully available (criterion; criterion description; category systems; category description, and category code) in the Supplementary Material. Table 1 shows its criteria and categories.

**Table 1:** Observation instrument: criteria and categories.

**Criterion**	**Category systems**
Participant	Participant 1
	Participant 2
	…
Recreational motor activity	Leaping hare
	Blind frog
	Jumping flea
	Flamethrower dragon
	Ball-catching dog
	Centipede wiping its feet
	Cunning fox
Specific motor skill	Locomotor skills
	Static Balance
	Dynamic Balance
	Propulsive skills
	Receptive skills
	Fine Motor Coordination
	Fine Motor Integration
Extremity	Right
	Left
Arm position	Backwards
	Forwards
	Across the body
	In the form of a cross with arms extended
	In the form of a cross with arms bent
	Others
Jump phase	Impulse
	Flight
	Landing
Leg position	Knees bent
	Knees not bent
Distance to the ground	Feet on the floor
	Heels lifted
	Feet in the air
Centimeters	Quartile 1 distance
	Quartile 2 distance
	Quartile 3 distance
	Quartile 4 distance
Base of support	Feet together
	Feet separated
	Feet widely separated
Type of landing	Without bouncing
	With a bounce
Precision of the jump	The 2 feet within the square
	At least one foot steps on a line of the square
	Outside of the square
Trunk position	Upright
	Inclined
Time	Quartile 1 time
	Quartile 2 time
	Quartile 3 time
	Quartile 4 time
Finger	Pinky finger
	Ring finger
	Middle finger
	Index finger
Direction	Direct
	Reverse
Part of the finger	Fingertip
	Other
Way of catching the ball	With both hands
	Supporting on body
	Not catching
Hand position	Together
	Separate
Height of the catch	Chest
	Neck head
	Abdomen
	Thighs
	Knee
	Under the knee
Attempt	1
	2
	3
	+3
Passing through	Transfer
	Touch
	Diverted
Shape orientation	Exact
	Inexact
Length of sides	Adequate
	Inadequate
Amplitude of angles	Appropriate
	Inappropriate
Intersection	Equal
	Unequal

#### Data Analysis Software

All analyses were carried out using IBM SPSS version 25 (IBM Crop, 2017).

### Procedure

The preschool management team was informed of the purpose, procedure, and benefits of the study. Once their approval was obtained, the parents were also informed and asked to complete the informed consent to authorize the participation of their children in the study.

Then, the parents that signed and delivered the informed consent were given an *ad hoc* questionnaire so that they could provide the information for the participant's exclusion criteria (having a history of pre, peri, or postnatal problems, neurological disease, sensory disturbance, mental or other clinically diagnosed impairment, or special needs), as well as information related to delivery mode, feeding type during the first 4 months of life, sex, and age. The preschool management team was given an *ad hoc* questionnaire to determine whether the potential participants complied with the first inclusion criterion: having been a student at the school since 1st year of Early Childhood Education.

Children who did not present exclusion criteria and who complied with the first inclusion criterion were assessed by the research team using the BADyG-I to verify their compliance with the second inclusion criterion: having a suitable IQ for their age. BADyG-I was administered following the instructions of its manual.

To observe the children's motor skills, the research team designed recreational motor activities, taking the following into account: (1) the study objective; (2) theoretical and empirical studies on childhood motor development (Hestbaek et al., 2017; Oberer et al., 2017; Goodway et al., 2019; Haywood and Getchell, 2019); (3) the Spanish Early Childhood Education curriculum, which determines the content related to motor skills to be worked on during this educational stage and the pedagogical resources to be used for it, being *play* especially highlighted (Education Science Ministry of Spanish Government, 2007); (4) spatial-temporal characteristics of the motor skill sessions carried out by the children during their regular school programming. Based on all of this, seven recreational motor activities were created, requiring the use of the gross and fine motor skills that are the subject of interest of this study (and previously defined in the Introduction Section). These skills are: locomotor skills; static balance; dynamic balance; receptive skills; propulsive skills (all referring to gross motor skills); visual-motor coordination and visual-motor integration (referring to fine motor skills). All of the recreational motor activities designed were accompanied by a brief fantasy-type story about animals, which was used to attract the children's attention, increase their motivation and encourage their engagement in the activities. This was done so since the imagination and fantasy, together with play, are the most common pedagogical resources used in Early Childhood Education (McLachlan et al., 2018), and animals are a common focus of attention in preschoolers (Born, 2018). Specifically, the seven recreational activities designed to promote the use the different motor skills were:

- Leaping hare: this game required the use of locomotor skills. From a specific point, the child was to jump with both feet together, as far as possible. When landing the jump, the child was unable to help using his/her hands, so the landing was made on foot. The child had three successive attempts (without recovery time) to do this.
- Blind frog: this recreational activity required static balance skills. The child was to remain as long as possible with his/her eyes closed, in a squatting position over the balls of the feet, keeping his/her body bent and the arms extended horizontally to the sides. If they lasted <5 s in this position, they could try again a second time (without recovery time).
- Jumping flea: this game involved dynamic balance. The child was to jump up and down without leaving a 25 cm square area, painted on the ground, looking forward (not at the ground).
- Flamethrower dragon: this game referred to propulsive skills. The child was to horizontally throw a tennis ball from the height of his/her shoulder so that it passed through a 30 cm diameter hoop that was 1.5 m away. They had to throw the ball 8 times (four successive throws with each hand and without recovery time between the throws made by each hand).
- Ball-catching dog: this game implied receptive skills. The child was to catch a ball thrown by an adult from a distance of 1.5 m. The adult made four successive throws.
- Centipede wiping its feet: this game entailed visual-motor coordination. Using their thumb, the child was to touch the fingertips of the other four fingers of the same hand. They were to do this successively, beginning with the pinky finger until reaching the index finger. Once touching this finger, they were to repeat these movements in reverse order, that is, from the index finger to the pinky finger. This series of movements was to be carried out once with each hand. They had three successive attempts to accomplish the task.
- Cunning fox: this game involved visual-motor integration, the child was to copy consecutively six shapes appearing on a sheet. The child could not review the figure to copy it. During the copying, the child could erase but not after the completion of the figure. The 6 figures to be copied were: a cross, triangle, square, arrow cross, rhombus, and triangle within another triangle.

The observation sessions were carried out in the school's motor development room, where the children's regular motor skills activities were carried out. Participants making up each class group attended the motor sessions at their regular time, together with their teacher, as usual. Before beginning each recreational motor activity, the teacher read the children the fantasy-based story corresponding to each activity. Visual instructions and a demonstration of each motor skill required were presented, maintaining the regular work method for the children and complying with the pedagogical guidelines indicated in the literature (Hamilton and Liu, 2018). The participants knew about this working method, but not the tasks, which were new to the participants; i.e., it was the first time that the participants performed these tasks. The tasks were carried out in five motor sessions that were developed on alternate days (respecting, as already indicated, the school schedule of the children's motor sessions). The tasks performed in each motor session were the following: 1st session, Blind frog; 2nd session, Ball-catching dog and 20 min later, Leaping hare; 3th session, Jumping flea and 20 min later, Centipede wiping its feet; 4th session, Cunning fox; and 5th session, Flamethrower dragon. This distribution of the tasks was carried out taking into account the usual duration of the motor sessions. The execution of each participant in each recreational motor activity was recorded for its subsequent observation and analysis.

These recordings were imported to the Lince software and were coded using the observation instrument (available in the Supplementary Material). An expert observer in observational methodology, Early Childhood Education, and motor development coded all of the observation sessions (301 sessions). Two months later, they were once again coded to calculate intra-observer reliability. A second observer, also an expert in these areas, coded all of the observation sessions to calculate the inter-observer reliability. To do so, the coded data were converted into a matrix of codes.

### Data Analysis

Data quality was calculated from a classic perspective that assessed the correlations arising in the categories of the observation instrument coded in each of the two recordings made by the first observer (intra-observer reliability), as well as the correlations between the categories coded in one recording of the first observer and those coded by the second observer (inter-observer reliability), based on the correlation coefficients of Pearson, Kendall's Tau-b, and Spearman. In addition, an index was sought out to relate to the association concept, using Cohen's Kappa.

To determine whether the variables of interest (delivery mode, feeding type during the first 4 months of life, sex, and age) influence motor skills, it was necessary to transform the observational data. For each participant, each category observed during the execution of each recreational motor activities was transformed into a score based on its degree of suitability for the execution of this activity, according to the literature on this area (Goodway et al., 2019; Haywood and Getchell, 2019). For each participant, the scores obtained in each activity were added. Thus, every participant obtained seven scores, each referring to one of the seven specific motor skills studied in this work: locomotor skills; static balance; dynamic balance; propulsive skills; receptive skills, visual-motor coordination, and visual-motor integration. Based on these scores, the following scores were also calculated: total balance score (total of the scores obtained on static balance and dynamic balance); total object control skills score (total of the scores on propulsive and receptive skills); total gross motor skills score (total of the scores on locomotor skills; static balance; dynamic balance; propulsive skills; and receptive skills); total fine motor skills score (total of the scores on visual-motor coordination and visual-motor integration); and total motor skills score (total of the scores on the 7 specific motor skills: locomotor skills; static balance; dynamic balance; propulsive skills; receptive skills, visual-motor coordination, and visual-motor integration). Therefore, each participant received a total of 12 scores.

To analyze whether there were differences in the motor skills based on delivery mode, the children's motor scores were grouped into two groups: those corresponding to children born via vaginal delivery and those of the children born via C-section.

To analyze whether there were differences in the motor skills based on feeding type during the first 4 months, the children's motor scores were grouped into two groups: one group made up of scores belonging to children who were exclusively breastfed and another group made up of scores for the rest of the children (those fed exclusively with formula + children receiving mixed-feeding), that is, those who received formula feeding to a greater or lesser extent. Given the sample size, it was impossible to create three groups based on the three types of feeding that were initially considered in the questionnaire. Therefore, and as with Tozzi et al. (2012), this classification was made based on two groups: exclusive breastfeeding and formula feeding.

To analyze whether there were differences in motor skills based on the participant's sex, their motor scores were grouped based on their sex, creating two groups: boys and girls.

To analyze whether there were an RAE on motor skills, the motor scores of the participants were grouped together into 2 groups based on the half of the year in which they were born -according to the grouping criteria used in past studies (Imbernón-Giménez et al., 2020; Martínez-Moreno et al., 2020)-: group 1 = children born during the last half of the year, that is from July 1 to December 31, who were the youngest participants. Their ages were between 63 and 68 months; group 2 = children born during the first half of the year, that is, from January 1 to June 30. These were the oldest participants. Their ages ranged from 69 to 74 months.

We calculated descriptive statistics in terms of group means (M) and standard deviations (SD). In all of the analyses of comparison of means, it was verified that the data followed a normal distribution through the Shapiro-Wilk test. In cases in which the data followed a normal distribution, a one-way ANOVA was used. In all other cases, for those not having a normal distribution, the Mann-Whitney U was used, although significant differences were never obtained with this test. All *p*-values lower than 0.05 (two-tailed) were considered statistically significant. For each of the differences obtained, the effect size was calculated using Cohen's *d* (Cohen, 1988), applied to the comparison of the means between groups, establishing the cut-off points of 0.00–0.19 = negligible; 0.20–0.49 = small; 0.50–0.79 = medium; and as of 0.80 = high.

## Results

The intra-observer agreement revealed a Kappa value = 0.99; a Pearson's r value = 1.00; Kendall's Tau b = 0.99 and Spearman = 0.99. The inter-observer agreement had a Kappa value = 0.92; a Pearson's r value = 0.99, Kendall's Tau b = 0.96 and Spearman = 0.97. In relation to Kappa intra-observer agreement, for each of the criteria, we obtained: Trunk position = 0.91 and for the rest of the criteria used, Kappa value = 1. The inter-observer Kappa value obtained for each of the criteria was: Attemp = 0.98; Arm position = 0.93; Distance to the ground = 0.91; Leg position = 0.91; Part of the finger = 0.89; Centimeters = 0.88; Hand position = 0.73; Trunk position = 0.69; and Height of the catch = 0.68. For the rest of the criteria, Kappa value = 1.

Therefore, the intra and inter observer reliability was found to be excellent, as was the quality of our observational data.

Significant differences were obtained in some of the motor skills measured based on delivery mode, type of feeding during the first 4 months of life, sex, and age.

Regarding the mode of delivery, children born by vaginal delivery were always found to have higher scores than children born via C-section (Table 2), except in Static balance. These differences were significant in: throwing [*F*_(1,33)_ = 4.56; *p* = 0.000, *d* = 0.63]; total control of objects [*F*_(1,32)_ = 9.57; *p* = 0.004, *d* = 0.97]; total gross motor skills [*F*_(1,31)_ = 8.99; *p* = 0.005, *d* = 0.95]; and total motor skills [*F*_(1,31)_ = 11.40; *p* = 0.002, *d* = 1.04].

**Table 2:** Means (M) and Standard Deviations (SD) of motor skill scores based on delivery mode.

**Motor skills**	**Vaginal delivery**		**C-section delivery**	
	**M**	**SD**	**M**	**SD**
Locomotor skills	18.05	3.15	17.46	2.87
Static balance	12.15	1.89	13.07	1.38
Dynamic balance	64.36	16.01	56.46	15.81
Throwing*	24.00	3.69	20.69	4.13
Catching	38.94	4.45	32.76	13.23
Visual-motor coordination	43.47	14.35	35.15	21.05
Visual-motor integration	7.36	2.16	5.61	1.32
Total balance	76.52	15.97	69.53	14.94
Total object control*	62.94	5.46	53.46	12.45
Total gross motor skills*	157.52	15.10	140.46	1.79
Total fine motor skills	50.84	14.38	40.76	20.22
Total motor skills*	208.36	21.28	181.23	23.79

* *Motor skill scores in which significant differences were detected, with p < 0.05*.

Regarding the type of feeding during the first 4 months of life (exclusively breastfeeding or formula-feeding), significant differences were found (with children who were exclusively breastfed obtaining the highest values) for the following: throwing [*F*_(1,32)_ = 6.48; *p* = 0.016, *d* = 0.75]; visual-motor integration [*F*_(1,34)_ = 33.44; *p* = 0.005, *d* = 0.94]; total control of objects [*F*_(1,31)_ = 11.88; *p* = 0.002, *d* = 1.02]; total gross motor skills [*F*_(1,30)_ = 5.77; *p* = 0.023, *d* = 0.82]; and total motor skills [*F*_(1,30)_ = 4.52; *p* = 0.042, *d* = 0.74] (Table 3).

**Table 3:** Means (M) and Standard Deviations (SD) of motor skills scores according to feeding type during the first 4 months of life.

**Motor skills**	**Exclusively breastfeeding**		**Formula feeding**	
	**M**	**SD**	**M**	**SD**
Locomotor skills	17.23	3.12	18.90	2.54
Static balance	12.61	1.77	12.36	1.74
Dynamic balance	63.04	15.40	57.54	17.71
Throwing*	23.76	3.65	20.54	4.39
Catching	39.00	4.67	31.54	13.83
Visual-motor coordination	41.00	16.12	38.36	20.72
Visual-motor integration*	7.33	1.98	5.36	1.50
Total balance	75.66	14.59	69.90	17.75
Total object control*	62.76	5.70	52.09	12.73
Total gross motor skills*	155.66	15.15	140.90	18.91
Total fine motor skills	48.33	16.13	43.72	6.07
Total motor skills*	204.00	23.66	184.48	25.94

* *Motor skill scores in which significant differences were detected, with p < 0.05*.

As for sex (Table 4), statistically significant differences were found (higher scores in boys) for throwing [*F*_(1,28)_ = 4.16; *p* = 0.041, *d* = 0.74] and total control of objects [*F*_(1,26)_ = 5.73; *p* = 0.024, *d* = 0.63]. Girls had statistically significant and higher scores on the following: static balance [*F*_(1,28)_ = 17.71; *p* = 0.000, *d* = 1.2]; visual-motor coordination [*F*_(1,30)_ = 6.02; *p* = 0.020, *d* = 0.79]; and total fine motor skills [*F*_(1,30)_ = 5.06; *p* = 0.032, *d* = 0.72].

**Table 4:** Means (M) and Standard Deviations (SD) of motor skills scores according to sex.

**Motor skills**	**Boys**		**Girls**	
	**M**	**SD**	**M**	**SD**
Locomotor skills	17.00	3.48	18.05	2.41
Static balance*	11.30	1.60	13.52	1.41
Dynamic balance	63.84	15.22	57.70	17.06
Throwing*	22.34	4.14	21.64	4.30
Catching	39.15	4.39	36.47	7.96
Visual-motor coordination*	33.38	16.34	47.58	14.96
Visual-motor integration	7.00	2.30	6.05	2.07
Total balance	75.15	15.50	71.23	16.40
Total control of objects *	64.00	6.39	58.11	8.68
Total gross motor skills	156.15	16.76	147.41	18.40
Total fine motor skills*	40.38	16.55	53.64	15.04
Total motor skills	196.53	26.52	201.05	25.19

* *Motor skill scores in which significant differences were detected, with p < 0.05*.

Regarding age (Table 5), statistically significant differences were found for dynamic balance [*F*_(1,41)_ = 5.08; *p* = 0.030, *d* = 0.66]; visual-motor integration [*F*_(1,43)_ = 4.78; *p* = 0.034, *d* = 0.63]; and total balance [*F*_(1,41)_ = 6.73; *p* = 0.013, *d* = 0.75]. In all cases, the higher scores were obtained by the group made up of the older children (aged 69–74 months), that is, those born in the first half of the year. Therefore, there were an RAE on the indicated motor skills.

**Table 5:** Means (M) and Standard Deviations (SD) of motor skills scores according to age.

**Motor skills**	**63–68 months**		**69–74 months**	
	**M**	**SD**	**M**	**SD**
Locomotor skills	18.38	2.81	17.55	2.95
Static balance	12.80	1.69	12.16	1.72
Dynamic balance*	58.00	16.83	69.27	10.82
Throwing	23.00	4.95	22.61	4.44
Catching	36.38	9.93	38.00	7.23
Visual-motor coordination	38.23	17.92	40.72	17.02
Visual-motor integration*	6.00	1.61	7.38	2.30
Total balance*	70.80	16.51	81.44	10.56
Total control of objects	59.38	11.53	60.61	7.67
Total gross motor skills	148.57	19.22	159.61	14.73
Total fine motor skills	44.23	17.85	48.11	17.20
Total motor skills	192.80	26.21	207.72	23.92

* *Motor skill scores in which significant differences were detected, with p < 0.05*.

## Discussion

This study has examined whether there were influences of delivery mode, feeding type during the first 4 months of life, sex, and age (more precisely, RAE) on motor skills (considered globally and specifically) evaluated in 5- and 6-year-old preschoolers. The results obtained suggest that this is a complex topic given that the influence of each of these variables on the studied motor skills is specific. In other words, their influence varies depending on the specific motor skills to be considered and depending on whether or not it is an overall score. Therefore, given that some (but not all) of the examined motor skills are found to be influenced by delivery mode, type of feeding during the first 4 months of life, sex, or age, it may be determined that two of the initially proposed hypotheses were corroborated (H_3_ and H_4_), while the other two being only partially supported (H_1_ and H_2_). It is difficult to make direct comparisons of these results with those from the literature, and it should be carefully done given the heterogeneity of the samples from each study (different ages, distinct socioeconomic and cultural contexts, etc.), the different motor skills studied, and the distinct activities/tasks and instruments used.

H_1_ affirmed that differences existed in the children's motor skills based on delivery mode. It was expected to find that children born via vaginal delivery would have higher motor skills than those born via cesarean section. The results indicate that not all of the motor skills revealed differences between the two types of children. Children born via vaginal deliveries displayed higher scores on: throwing, total object control, total gross motor skills, and total motor skills; there was a medium or large effect size in all of the cases. For the remaining motor skill scores, no significant differences were found. Therefore, only some gross motor skills, no fine motor skills, as well as total motor skills were found to be influenced by delivery mode. These results support the findings of past studies such as those by Rebelo et al. (2020), who also found that the influence of delivery mode on motor skills varied depending on the type of motor skill considered. Our results are coherent with those of these authors, as they suggest differences favoring children born by vaginal delivery for object control skills and total motor skills, and no difference for locomotor and balance skills. However, unlike the results found by these authors, we have not found an influence of delivery mode on visual-motor coordination, visual-motor integration, or total fine motor skills (all referring to fine motor skills). Similarly, our results vary from those found by Blazkova et al. (2020), who also found differences in visual-motor integration based on the children's delivery mode. Considering our results, and unlike those of other studies (Grace et al., 2017), it is not possible to absolutely declare that being born by C-section will result in poorer motor skills. However, it may be suggested that its influence appears to be specific to certain motor skills. The discrepancies arising between studies may be due not only to the previously mentioned variables (different sample characteristics, motor skills, tasks, and instruments used) but also to the classification of the delivery modes that was used in each study. Therefore, in our study, even using a classification that has been previously used in the literature, there was no differentiation made as to whether the vaginal delivery involved the use of instruments or not (for example, the use of forceps, vacuum, and spatulas), or whether the C-section was programmed or due to an emergency. Some authors have indicated that these aspects, not considered in our work, may have distinct effects on children's motor development (Tribe et al., 2018; Takács et al., 2020).

As for H_2_ (referring to the existence of differences in the children's motor skills based on the type of feeding received during the first 4 months of life, it was expected to find that children that were exclusively breastfed during this period would have better motor skills than those fed with formula or via a mixed-feeding mode). The results indicate that only some of the motor skills presented differences based on feeding type (although the effect size was always medium or large). These skills are: throwing; visual-motor integration; total control of objects; total gross motor skills, and total motor skills, with the children that were exclusively breastfed obtaining the higher scores. For the rest of the motor skills analyzed, there were no statistically significant differences found. These results are distinct from those of Bellando et al. (2020) and Michels et al. (2017), who did not find any effect of feeding type on motor skills beyond the 3 first months of life. Our results are along the lines of those of Jardí et al. (2018) and Kádár et al. (2021) since we found that the influence of infant feeding type on motor skills continue for longer periods of time. Furthermore, like Dee et al. (2007), this influence is found for some gross and some fine motor skills. In our study, more gross motor skill scores (4) than fine ones (1) were influenced by feeding type, allowing us to conclude that gross motor skills appear to be more sensitive to the influences of feeding type than fine motor skills, unlike the findings of Leventakou et al. (2015).

Once again, these differences may be due to distinct factors. In addition to those mentioned above, the feeding time period that participants were asked about in the distinct studies should be considered. As explained above, in our study, parents were asked about the feeding type for the first 4 months of life, given that, in Spain, this is when maternal leave ends and mothers tend to go back to work, often deciding to no longer breastfeed (Jardí et al., 2018; Cabedo et al., 2019). Therefore, asking about the feeding type beyond these first 4 months of life would probably not have resulted in the creation of a group of children that were exclusively breastfed. Other studies on breastfeeding and infant feeding, carried out in other countries (not Spain), also used this time period as the turning point in infant feeding (Michels et al., 2017). However, other works have used a cut-off point of 6 months (Grace et al., 2017; Kádár et al., 2021). This temporal difference may contribute to the distinct results found among the different studies. Some works have also considered the frequency of feeding (how often a child was breastfed or how much milk drank each day) (Khan et al., 2019), an issue that may also lead to the variable results of the literature.

As for the influence of sex on the children's motor skills (H_3_), as we hypothesized, the results indicate that boys outperformed girls on certain skills (throwing and total object control) while girls outperformed boys on other motor skills (static balance; visual-motor coordination and total fine motor skills). In all of the cases, the effect size was medium or large. Also in line with the hypothesis, for certain motor skills (the remaining motor skills studied), no significant differences were found between both sexes. Our results are coherent with those found by other authors who also failed to detect differences in locomotor skills (Bakhtiar, 2014; Foulkes et al., 2015; Barnett et al., 2016; Bolger et al., 2018, 2020), and who found better object control skills in boys (Foulkes et al., 2015; Barnett et al., 2016; Venetsanou and Kambas, 2016; Kokstejn et al., 2017; Bolger et al., 2018, 2020; Mecías-Calvo et al., 2021). It should be noted that other studies have obtained distinct results, defending the existence of differences in locomotor skills between genders, in favor of boys (Robinson, 2010), or girls (Bolger et al., 2018, 2020; Wang et al., 2020), or no differences in object control (LeGear et al., 2012; Bakhtiar, 2014). As for balance skills, our results have demonstrated a better static balance in girls, but no difference between both sexes in dynamic balance and total balance. The lack of a difference between both sexes in total balance is in line with the findings of other studies (Singh et al., 2015; Barnett et al., 2016) although it contradicts others that found higher scores in girls (Venetsanou and Kambas, 2016; Kokstejn et al., 2017; Mecías-Calvo et al., 2021). The remaining results referring to the balance skills (better static balance in girls and no differences in dynamic balance), cannot be compared with past works since the studies do not differentiate between both types of balance skills, offering an overall score on balance skills. Therefore, our work offers additional information to overcome this information deficiency in the literature regarding specific balance skills of preschoolers.

Our results also offer novel information on the influence of sex on specific preschool fine motor skills, given that there was a gap in the literature regarding this issue. Our results indicate higher scores for girls on visual-motor coordination and total fine motor skills and a lack of differences in visual-motor integration. Therefore, it may be concluded that sex appears to distinctively influence motor skills, when considered both globally and specifically. According to many authors, these differences between girls and boys are not necessarily due to their physical characteristics (since before puberty, they are quite similar) but rather, they may be caused by the distinct experiences of boys and girls participating in different activities. This may be related to gender stereotypes, often promoted by parents and teachers (Bolger et al., 2020; Matarma et al., 2020). Therefore, girls tend to be more likely to participate in cultural and artistic activities (painting, drawing, handicrafts, or playing an instrument, which are more related to fine motor skills) and are less likely to be involved in sporting activities (more associated with gross motor skills). When they participate in physical and sports activities, they tend to be those such as ballet (associated with balance) as opposed to ball sports such as soccer or tennis (related to object control) (Hernández Luengo et al., 2019; Bolger et al., 2020; Matarma et al., 2020). However, co-education and gender equality policies are becoming increasingly frequent in our country (Venegas et al., 2019), which may explain why, in our study, there was a larger number of motor skills in which no differences were found based on sex, as compared to those in which differences did indeed exist.

H_4_ refers to the RAE on preschool motor skills. We hypothesized that children born during the first half of the year would outperform those born during the second half of the same year on certain motor skills. Our results corroborate this hypothesis. Children born during the first half of the year, that is, the older children, displayed better visual-motor integration, dynamic balance, and total balance, with a medium to large effect size in all of the cases. No differences were found in the remaining motor skills examined. These results support some of the results found in the literature but they contradict others. While some other authors also found an RAE on balance (Mecías-Calvo et al., 2021) and on visual-motor integration (Imbernón-Giménez et al., 2020), other studies have not confirmed the existence of differences in balance (Imbernón-Giménez et al., 2020; Navarro-Patón et al., 2021). In our study, as in other works, no differences were found in total object control (Imamoglu and Ziyagil, 2017; Mecías-Calvo et al., 2021) or total motor skills (Navarro-Patón et al., 2021). Other works contrast with these results (the existence of differences in object control: Imbernón-Giménez et al., 2020; Navarro-Patón et al., 2021; and in total motor skills: Mecías-Calvo et al., 2021). Our study also failed to find an RAE on visual-motor coordination, unlike other studies (Mecías-Calvo et al., 2021; Navarro-Patón et al., 2021). It also did not find an RAE on locomotor skills. As for the latter, Imamoglu and Ziyagil (2017) found differences in some of these, but not in others, suggesting a great specificity of the RAE on motor skills, since even within one type of motor skills, such as locomotor, depending on the specific task or activity being analyzed, the results may vary. Therefore, as mentioned above, the distinct tasks used to assess the motor skills in the diverse studies make the direct comparison of results quite difficult (De Niet et al., 2021) and may contribute to the variety of results in the literature. We are unaware of studies that have analyzed the RAE on the remaining specific motor skills considered in our study: throwing, catching, static balance, and dynamic balance, aspects in which, except for the latter, we have not detected an RAE.

To conclude, the distinct motor skills analyzed reveal distinct degrees of sensitivity to the influence of delivery mode, infant feeding type, sex, and RAE. Vaginal delivery, having been exclusively breastfed for the first 4 months of life, and being older than one's peers (as opposed to being born via C-section, having been formula-fed, and being younger than one's classmates) are characteristics that appear to favor certain motor skills. Although not all of the motor skills are positively influenced by these aspects, no motor skills are negatively influenced by them. Sex influences some (but not all) motor skills, with boys outperforming girls for some skills, and the opposite being found for others.

The specificity of the results obtained suggests the need to design individualized interventions aiming at improving the motor skills which may be at risk for each child, based on their present (such as sex and age) and past (such as delivery mode and feeding type) characteristics. According to other authors, this series of results allows us to conclude that certain biological events (such as sex and age), and some experiences in very early life (such as delivery mode and type of feeding during the first 4 months), are especially influential on preschool motor skills and that the influences of some very early experiences on human development may be evident even years later (Nelson and Gabard-Durnam, 2020).

Although these results should be carefully considered due to the limitations of this study (see below), they may be quite relevant, given is the current lack of knowledge on preschool motor skills (Imbernón et al., 2021). This study attempts to fill this gap, providing information on the influence of certain factors on these skills, an essential aspect to design effective interventions that respond to the distinct needs of children. It should be noted that a highlight of our study is the analysis of the influence of four factors on these motor skills. In the majority of studies of this type, only one factor is considered (Barnett et al., 2016). Therefore, our study offers information that may be of great interest as it permits a deeper understanding of preschool motor skills. It is especially relevant and useful for teachers, other professionals, and researchers working with children in healthcare, educational, social, or sporting environments. Our results should also be considered by policymakers, given that they suggest the need to implement public policy strategies aiming to improve children's motor skills and that would, thereby, promote a physically active and healthy lifestyle. This will be considered in greater detail below.

As for the contributions and implications of this study on daily teaching practices, we consider that the information about the recreational motor activities and the assessment process of motor skills, as well as the observation instrument offered, can be very useful. This is even more so if we consider that: (1) Education Science Ministry of Spanish Government (2007) and other international institutions (Early Head Start National Resource Center, 2013) indicate that preschooler development and learning must be assessed using direct and systematic observation; and (2) many early childhood teachers recognize their lacking of knowledge, skills, and resources in the motor assessment field (Cueto et al., 2017). Therefore, we believe that the detailed and extensive assessment process of preschool motor skills conducted via systematic observation is another strong point of this work. This suggests that the assessment of motor skills was: (1) objective –not subjective as some teachers had admitted to being (Cueto et al., 2017)– and was not based on third party information, as it often occurs in studies (Khalaf et al., 2015; Takács et al., 2020), despite the limitations that this may imply (Blanco-Villaseñor and Escolano-Pérez, 2017); (2) carried out in the child's natural setting, such as at school, capturing the spontaneous motor execution by the children during recreational activities that are significant and of interest to them. This assessment is characterized by a high ecological validity (Blanco-Villaseñor and Escolano-Pérez, 2017), and allows us to overcome the ecological validity issues present in other studies on motor skill assessment (Tamplain et al., 2020); and (3) carried out using an instrument created based on the objective and context of the study. In other words, not using an instrument created from a clinical perspective, like the majority of the tools intended for the assessment of children's motor skills and which, despite their limitations, are often used in studies carried out in scholastic contexts (Lindsay et al., 2018; Klingberg et al., 2019; Morley et al., 2019). It should be noted that, despite the previously mentioned relevance of Early Childhood Education for the appropriate development and learning of children's motor skills, the literature highlights a lack of instruments available to assess said skills in the educational environment (Klingberg et al., 2019; Morley et al., 2019). Our study, and specifically the observation instrument offered, which is also free of charge, contributes to eliminating this gap. This instrument also overcomes the limitations of the instruments for motor skill assessment developed from a clinical perspective, which are of extended use without considering the context in which the assessment is performed [such as, the Motor Assessment Battery for Children-2 (MABC-2), the Motor-Proficiency-Test (MOT4-6), or the Test for Gross Motor Development-2 (TGMD-2)]: (1) These instruments require specific materials, unavailable in the school setting (Platvoet et al., 2018); (2) In these instruments, aimed at the assessment of children with risks or difficulties, the spectrum of levels of the motor skills assessed tends to be limited. Consequently, they do not allow for the determination of the large variability of skills that may be demonstrated by children with more typical development, or even the levels of motor skills that may be demonstrated by children with more advanced or highly stimulated development (Klingberg et al., 2019; Morley et al., 2019). The observation instrument used in this study overcomes these limitations since: (1) it does not have equipment requirements; (2) it permits the assessment of a broad spectrum of motor skill levels (from low to high performance), which are also considered at both a global and specific level. This is noteworthy since many motor skill assessment instruments only permit the evaluation of specific motor skills, but not all the skills that have been identified on a theoretical level. Thus, many motor skill assessment instruments reveal incoherencies between the theoretical and practical aspects. Although at a theoretical level this classification of gross and fine motor skills is widely accepted, along with the differentiation of various specific motor skills within each category, most assessment instruments do not reflect this structure, being restrictive and insufficient to assess the large set of abilities making up the motor skills (De Niet et al., 2021). Furthermore, this instrument, according to the recommendations of the most recent literature (Palmer et al., 2021), considers process-oriented motor skills assessment (how a movement is performed) and product-oriented assessment (the outcome of a movement). To date, product-based measures are the most common (Chang et al., 2020), with very few studies using both product- and process-based assessments to measure preschool motor skills (Szeszulski et al., 2021). Our study has addressed this gap. Despite considering both approaches, the process-approach is more important in our instrument, since this approach provides more useful information so that teachers can provide instructional skill-specific feedback to the children on their performance, a necessary element for educational practices aimed at the development of motor skills to be more effective (Bolger et al., 2020).

Ultimately, all of the characteristics that define our instrument make it useful and appropriate so that teachers may perform an objective, thorough, and profound assessment of the preschoolers' specific gross and fine motor skills. Based on this information, educational practices responding to the needs of each child may be designed.

All this issues can be also interesting to researchers given comprehensive assessment of children's motor skills is a significant concern in the contemporary child motor research field (Chang et al., 2020).

Our results suggest the need to develop public health, social and educational policies that promote infant motor skills. Therefore, in clinical practice, it is necessary to raise awareness so that obstetricians adopt the World Health Organization (2018) recommendations to reduce unnecessary cesarean sections and to develop high-quality antenatal education programs that offer information to parents on the effects of cesarean delivery, to avoid C-sections by demand. The same should occur with regard to feeding. In addition to informing parents as to the benefits of breastfeeding, a social environment should be created to favor it. Social policies should be implemented and facilities should be offered to promote this practice, such as increased maternity leave or the creation of breastfeeding rooms at work and social sites. Our results also suggest the need to reflect on the organizational policies of the school system, given the RAE detected on certain motor skills. Grouping students based on the half-year in which they were born, and not based on the entire year, would result in more similar levels of motor skills for children attending the same class, thus promoting a more beneficial educational experience for all.

Certain study limitations should be considered. The information referring to the mode of delivery and type of feeding during the first 4 months of life was collected retrospectively from the parents, therefore, some recall bias may have taken place. However, the retrospective collection of these type of data is a widely used resource in the literature to obtain perinatal data and to characterize child development histories (Khalaf et al., 2015; Bornstein et al., 2020), given the difficulty (and even impossibility) of obtaining data from medical or other professional records. Some authors indicate that the validity and reliability of parental recall in data collection are assured when the data are collected within 1–3 years after the relevant event took place (Grace et al., 2017). Other authors extend this time up to 6 years (Keenan et al., 2017), or even up to 20 years, after the event (Natland et al., 2012). According to these authors (Natland et al., 2012; Keenan et al., 2017), we can consider the information provided in this study by the parents to be valid and reliable.

Some authors have indicated that the validity and reliability of parental recall are affected by aspects such as the specificity of the considered event (Bornstein et al., 2020). To facilitate and increase the validity and reliability of the parental recall, as mentioned previously, in the study, parents were asked about general aspects of the delivery, specifically, whether it was vaginal or cesarean, without requesting more detailed information. In the future, it would be interesting to collect as much information as possible about other more specific issues of vaginal/cesarean delivery (for example, instrument use or not during the vaginal delivery), although this implies assuming a greater risk regarding potential parental recall bias.

A similar situation is found for feeding type. As previously mentioned, in our study, no information was collected on the frequency or duration of breastfeeding or the type of feeding used after the first 4 months, aspects which may also affect the children's motor skills (Khan et al., 2019). In the questionnaire administered to the parents, response options did not include the option of providing breast milk in a bottle, another possible feeding type. However, none of the parents indicated this in the “considerations” section of the questionnaire. These limitations may be interesting to consider in future studies.

It should also be considered that the study carried out is a punctual design. Therefore, in the future, it may be interesting to carry out a follow-up study to determine whether changes are found in the influence of the variables studied here regarding the distinct motor skills as the children grow up.

Another aspect to be considered is the small sample size and its non-random nature. It should be noted, however, that observational studies do not seek the representativeness of the sample, but rather its intensive study. There is a greater interest in obtaining a large quantity of detailed information on the natural behavior of a small number of participants than in the representativeness with respect to a larger population (Anguera, 2003). Nevertheless, in the future, it may be interesting to increase the number of participants in the study, which would also assist in the analysis of the influence on motor skills of each of the 3 infant feeding types considered in the questionnaire. However, it should be taken into account that, given the participants are minors, and the assessment of their motor skills is carry out with observational methodology, increasing the sample size may result in great complexity, effort, and dedication (Salamon, 2017; Maddox, 2019). Therefore, before increasing the sample size, it may be interesting to conduct an analysis of generalizability and an optimization plan to assess costs/benefits (Blanco-Villaseñor and Escolano-Pérez, 2017).

Another limitation of this study is its failure to consider potential confounders. This is a common limitation in this type of studies given the complexity of motor skills and their development (Hernández Luengo et al., 2019). Therefore, in the future, it may be interesting to also consider the effect of potential interactions between the variables analyzed in this study on the motor skills, including other potential variables that have not been considered and which, apart from, or in addition to, the factors considered, may also affect the children's motor skills. These variables may refer to both the child (anthropometric measurements such as weight, body mass index; type of activities –sports and others– carried out in their free time; etc.) and the parents (mother's age at birth, parents' education, smoking during pregnancy, etc.), as well as the family context (quality of home stimulation received, presence of older siblings acting as models for developing motor skills, etc.) and the social context in which the child develops (such as proximity and ease of access to sporting installations). Numerous factors may influence childhood motor skills. Although it was not within the scope of this study to consider every variable in the analyses, they should be carefully considered when interpreting the results of our study and conducting further research on the area, given the complexity. In the future, interdisciplinary collaborations will be necessary to better understand how and why these and other potential factors have specific influences on motor skills.

## Conclusion

Preschool motor skills are a complex topic. They show distinct degrees of sensitivity to different early environmental and biological variables such as delivery mode, type of feeding during the first 4 months of life, sex, and age. More exactly, vaginal delivery, having been exclusively breastfed for the first 4 months of life, and being older than one's peers (as opposed to being born via C-section, having been formula-fed, and being younger than one's classmates) favor certain (not all) preschool motor skills. No motor skills are negatively influenced by them. Sex influences some (but not all) motor skills, with boys outperforming girls for some skills, and girls outperforming boys for others.

Very important practical implications for teachers, other professionals, and researchers working with children in healthcare, educational, social, or sporting environments are derived from these results. Our results should also be considered by policymakers, given that they suggest the need to implement public health, social and educational strategies aiming to improve children's motor skills and that would, thereby, promote a physically active and healthy lifestyle.

## Data Availability Statement

The raw data supporting the conclusions of this article will be made available by the authors, without undue reservation, to any qualified researcher.

## Ethics Statement

The studies involving human participants were reviewed and approved by The Research Unit of the University of Zaragoza. The research was also approved by the school management team. In accordance with Organic Law 15/1999 of December on the Protection of Personal Data (1999, Official State Gazette no. 298, of December 14), all parents of the participants signed the informed consent authorizing their children's participation in the study and the recording of the children. Furthermore, and following the guidelines of the aforementioned law, observers signed a confidentiality agreement. No special ethical approval was required for this research since the Spanish public education system and national regulations do not require such approval. Each participant received a small reward (two chocolates) in gratitude for their participation. Written informed consent to participate in this study was provided by the participants' legal guardian/next of kin.

## Author Contributions

EE-P was involved in conceptual and methodological structure, literature review, data collection, systematic observation, manuscript drafting, and discussion. CS-L was involved in methodological structure and data analysis. MH-N was involved in data collection and systematic observation. All of the authors contributed to revising the manuscript and provided final approval of the version to be published.

## Conflict of Interest

The authors declare that the research was conducted in the absence of any commercial or financial relationships that could be construed as a potential conflict of interest.

## Publisher's Note

All claims expressed in this article are solely those of the authors and do not necessarily represent those of their affiliated organizations, or those of the publisher, the editors and the reviewers. Any product that may be evaluated in this article, or claim that may be made by its manufacturer, is not guaranteed or endorsed by the publisher.
