# Supplementary material for: Early Environmental and Biological Influences on Preschool Motor Skills: Implications for Early Childhood Care and Education
Source: Front Psychol. 2021 Aug 13;12:725832. doi: 10.3389/fpsyg.2021.725832 (PMC8414646; doi:10.3389/fpsyg.2021.725832)
Supplement: Supplementary file 1 [file Table_1.pdf]

Criterion	*Criterion description*	*Category systems*	*Category description*	*Category code*
Participant	Child carrying out the motor activity.	Participant 1	Participant 1 carries out the motor activity.	P1
		Participant 2	Participant 2 carries out the motor activity.	P2
		...	The participant... carries out the motor activity.	Pn
Recreational motor activity	Playful activity or task performed by the participant.	Leaping hare	Activity in which the participant, situated on a specific point, jumps forward with both feet together, propelling themselves with their arms. The participant should land on his/her feet, not touching the ground with his/her hands.	Sl
		Blind frog	Activity in which the participant, with eyes closed, should remain in a squatting position over the balls of his/her feet, which should be separated by approximately 30 cm, with his/her body bent and arms extended horizontally to the sides (that is, extended in the form of a cross).	C
		Jumping flea	Activity in which the participant, situated on a point that is the center of a square measuring 25 cm per side, and looking forward, should jump up and down ten times in a row, landing on the same point where he/she began.	Ss
		Flamethrower dragon	Activity in which the participant should throw a tennis ball horizontally so that it passes through a hoop that is situated at a distance of 1.5 meters away from them.	Lh
		Ball-catching dog	Activity in which the participant should catch a ball that is thrown by an adult from a distance of 1.5 meters.	Rp
		Centipede wiping its feet	Activity in which the participant should use their thumb to touch the fingertips of the other fingers of the same hand, one at a time, first touching the pinky finger, and continuing in reverse order.	Td
		Cunning fox	Activity in which the participant should copy 6 shapes of distinct complexities: cross, triangle, square, arrow cross, rhombus, triangle within another triangle.	Cf
Specific motor skill	Specific motor skill carried out by the participant.	Locomotor skills	Actions of large muscle and postural groups that involve the coordination of the entire body, permitting the body's transfer from one point in space to another, through body movement.	Ls
		Static Balance	The ability to hold a position during stationary tasks such as standing or sitting.	Sb
		Dynamic Balance	The ability to hold a position during activities requiring movement, such as walking. It is obtained when the body's stability is maintained during movement performance.	Db
		Propulsive skills	The ability to send an object away from the body.	Ps
		Receptive skills	The ability to receive an object.	Rs
		Fine Motor Coordination	It refers to small muscle movements but not to the integration of these muscle movements with other input, such as visual-spatial information, from the environment.	Cmf
		Fine Motor Integration	Organization of small muscle movements in the hand and fingers with the processing of visual stimuli. Visual information from the environment must be processed and integrated with fine motor.	It
Extremity	Upper or lower limb (depending on the activity at hand) with which the participant carries out the movement. It applies to the following activities: *Flamethrower dragon* and *Centipede wiping its feet*.	Right	The (upper or lower) right limb is used by the participant to carry out the movement.	ED
		Left	The (upper or lower) left limb is used by the participant to carry out the movement.	EIz
Arm position	Posture in which the arms are positioned while the participant performs the action. It applies to the following activities: *Blind frog*, *Leaping hare* (to each of the jump phases) and *Flamethrower dragon.*	Backwards	The arms are placed behind the participant's shoulders while he/she performs the action (which may be a throw from the back to the front or another action with the arm).	PbA
		Forwards	The arms are placed in front of the participant's shoulders while he/she performs the action, without having been, at any time, behind them during the course of the action.	PbD
		Across the body	The arms are extended outwards, following the directionality of the participant's body while he/she performs the action.	PbC
		In the form of a cross	Upon performing the activity, the participant keeps his/her arms extended horizontally in the form of a	PbCzE

		with arms extended	cross.	
		In the form of a cross with arms bent	Upon performing the activity, the participant keeps his/her arms horizontally bent in the form of a cross.	PbCzFl
		Others	Upon performing the activity, the participant keeps his/her arms in a position that differs from those mentioned above.	PbO
Jump phase	One of the three phases that may be distinguished in the jump, that is, one of the three moments that can be distinguished in the movement produced by the action of one or both legs, through which the participant's body leaves the ground as a support surface, later returning to it. This applies to the *Leaping hare* activity.	Impulse	Jump phase where the trajectory to be followed by the participant is determined. It requires a bending, followed by a quick extension of the lower trunk.	FsIm
		Flight	Jump phase in which the participant loses contact with the surface. It implies a horizontal displacement across the air.	FsV
		Landing	Jump phase in which the participant comes into contact with the ground once again. In this phase, the accumulated energy is absorbed or transformed.	FsR
Leg position	Space between the ground and the participant's feet. This applies to the following activities: *Leaping hare* (in each of the phases of the jump) and *Ball-catching dog.*	Knees bent	While performing the activity, the participant keeps his/her knees bent, so that his/her abductor muscles and calves are close together.	PRfl
		Knees not bent	While performing the activity, the participant keeps his/her knees extended, without bending them.	PRsfl
Distance to the ground	Space between the ground and the participant's feet. This applies to the following activities: *Leaping hare, Blind frog* and *Jumping flea*. In the *Leaping hare* activity, it is applicable to each of the jump phases.	Feet on the floor	The balls of the participant's feet are kept fully on the ground. (In the *Jumping flea* activity, this only applies to the landing of the jump phase).	DsPs
		Heels lifted	Only the balls of the participant's feet touch the ground, with their heels being lifted up from the ground.	DsTe
		Feet in the air	The participant's feet are in the air, not touching the ground with any part of them. This category is not applicable to the *Blind frog* activity.	DsPa
Centimeters	Space, distance measured in cm from the point where the propelling takes place, as done by the participant when jumping, to the point where the landing of said jump occurs. This only applies to the *Leaping hare* activity.	Quartile 1 distance	The participant has jumped a distance that is situated between 0 and 25% from the lowest distances jumped by the study participants.	C1
		Quartile 2 distance	The participant has jumped a distance that is situated between 26% and 50% from the lowest distances jumped by the study participants.	C2
		Quartile 3 distance	The participant has jumped a distance that is situated between 51% and 75% from the greatest distances jumped by the study participants.	C3
		Quartile 4 distance	The participant has jumped a distance that is situated between 76% and 100% from the greatest distances jumped by the study participants.	C4
Base of support	Space, distance between the participant's feet. This applies to the following activities: *Leaping hare* (to each of the jump phases), *Jumping flea* (to the landing of the jump) and *Flamethrower dragon*.	Feet together	The participant's feet touch one another.	BsPj
		Feet separated	The participant's feet are separated by a distance of less than 15 cm.	BsPs
		Feet widely separated	The participant's feet are separated by a distance of more than 15 cm.	BsPms
Type of landing	Means in which the landing of the jump takes place in the *Leaping hare* activity. This only applies to this activity.	Without bouncing	During the landing phase of the jump in the *Leaping hare* activity, the participant keeps his/her feet immobile with respect to the first position in which he/she landed. It is considered a correct jump.	TcSr
		With a bounce	During the landing phase of the jump in the *Leaping hare* activity, the participant moves his/her feet with respect to the first position in which he/she landed. It is considered an incorrect jump.	TcCr
Precision of the jump	In the *Jumping flea* activity, adjustment between the position of the feet in the jump landing phase and the square that is painted on the ground (participant location at the start of the activity). That is, this category assesses if the participant, during each jump, lands in the same area where he/she began the task.	The 2 feet within the square	During the landing phase of the jump, the participant lands with the 2 feet within the square, without stepping on any of its lines.	Ps2Dt
		At least one foot steps on a line of the square	During the landing phase of the jump, the participant lands stepping on at least one of the lines of the square.	Ps1Dt
		Outside of the square	During the landing phase of the jump, the participant lands with at least one foot completely outside of the square.	PsF
Trunk position	Position adopted by the torso. It applies to the following activities: *Blind frog, Flamethrower dragon* and *Ball-catching dog*.	Upright	The participant's torso is positioned vertically with respect to the ground, that is, their torso is at a 90º angle to the ground	PtE
		Inclined	The participant's torso is not vertical with respect to the ground, that is, the participant's torso forms an	PtI

			acute or obtuse angle with the ground.	
Time	Seconds in which the participant carries out the activity. This applies to the *Blind frog* activity.	Quartile 1 time	The time that the participant spends performing the activity falls within quartile 1 of the times used by all of the participants.	T1
		Quartile 2 time	The time that the participant spends performing the activity falls within quartile 2 of the times used by all of the participants.	T2
		Quartile 3 time	The time that the participant spends performing the activity falls within quartile 3 of the times used by all of the participants.	T3
		Quartile 4 time	The time that the participant spends performing the activity within quartile 4 of the times used by all of the participants.	T4
Finger	Finger touched by the participant with his/her thumb. This applies to the *Centipede wiping its feet* activity.	Pinky finger	Three-phalange finger, located on the external side of the hand.	DM
		Ring finger	Three-phalange finger, located next to the pinky finger.	DAn
		Middle finger	Longest three-phalange finger, located in the center of the hand.	DCo
		Index finger	Three-phalange finger, located next to the thumb, between this and the middle finger.	DIn
Direction	Direction or order in which each finger is touched within the touching sequence. This only applies to the *Centipede wiping its feet* activity.	Direct	Starting with the pinky finger, the participant touches the other fingertips until reaching the index finger.	SDi
		Reverse	Starting with the index finger, the participant touches the other fingers until ending with the pinky finger.	SInv
Part of the finger	Part of the finger that is touched by the thumb. This only applies to the *Centipede wiping its feet* activity.	Fingertip	The participant touches one of his/her fingertips with his/her thumb. That is, the participant's thumb touches a finger on its upper part, on the opposite side of the nail.	PdYe
		Other	The participant touches another finger with his/her thumb on any of its parts other than the fingertip.	PdOt
Way of catching the ball	Way in which the participant catches the ball that has been thrown, using or not using his/her body to help. This only applies to the *Ball-catching dog* activity.	With both hands	The participant catches the ball with both arms and hands held forward and separated from his/her body, holding it between his/her hands.	Rp2m
		Supporting on body	The participant uses his/her body to catch the ball, supporting the ball with their body.	RpAc
		Not catching	The participant does not catch the ball so that it falls to the ground, even if it was previously touched with the hands or another body part, or even when it was previously caught for a brief period of time. (That is, this category is coded regardless of whether the participant momentarily trapped the ball but it immediately fell out of his/her hands).	RpNr
Hand position	Posture adopted by the participant's hands when catching the ball. This only applies to the *Ball-catching dog* activity.	Together	The participant catches the ball with both hands touching each other, with their palms facing upward.	PmJ
		Separate	The participant catches the ball with his/her hands separated, situated one on each side of the ball.	PmS
Height of the catch	Vertical distance at which the ball is recovered with respect to the ground. This only applies to the *Ball-catching dog* activity.	Chest	The participant catches the ball at chest height.	ArP
		Neck head	The participant catches the ball at face or neck height.	ArCc
		Abdomen	The participant catches the ball at abdomen height.	ArAb
		Thighs	The participant catches the ball at thighs height.	ArM
		Knee	The participant catches the ball at knees height.	ArRd
		Under the knee	The participant catches the ball when it is below his/her knees.	ArDrd
Attempt	A participant's attempt to perform the task. This applies to the following activities: *Leaping hare, Blind frog, Centipede wiping its feet* and *Cunning fox*.	1	The participant performs the task for the first time.	I1
		2	The participant performs the task for the second time.	I2
		3	The participant performs the task for the third time.	I3
		+3	The participant performs the task for the fourth time, or more.	Im3
Passing through	It indicates whether or not the ball passes through the hoop. This applies to the: *Flamethrower dragon* activity.	Transfer	The ball passes through the hoop.	AtEc
		Touch	The ball touches the hoop but does not pass through it.	AtTn
		Diverted	The ball is diverted and does not pass through or touch the hoop.	AtN
Shape orientation	Spatial location of the shape copied by the participant. This applies to the *Cunning fox* activity.	Exact	The shape copied by the participant is spatially located in the same way as the shape appearing in the model.	OEx

		Inexact	The shape copied by the participant is spatially located in a different way to the shape appearing in the model.	OI
Length of sides	Length of the sides making up the shape copied by the participant. This applies to the *Cunning fox* activity.	Adequate	The length of the sides of the shape copied by the participant is equal to that of the sides of the stimuli presented as a model.	LdAd
		Inadequate	The length of the sides of the shape copied by the participant is different from the length of the sides of the stimuli presented as a model.	LdIn
Amplitude of angles	Amplitude of the angles (separation distance between the two sides of an angle) making up the shape copied by the participant. This applies to the *Cunning fox* activity.	Appropriate	The amplitude of the angles of the shape copied by the participant is equal to the angles of the shape appearing as a model.	AaAp
		Inappropriate	The amplitude of the angles of the shape copied by the participant is different from the angles of the shape appearing as a model.	AaAId
Intersection	Intersection points or union of the lines making up a stimulus. This applies to the stimuli for the *Cunning fox* activity.	Equal	The points of intersection or union of the elements making up the stimuli copied by the participants are situated at the same positions as those of the model.	Ii
		Unequal	The points of intersection or union of the elements making up the stimuli copied by the participants are situated at different positions as those of the model.	Id
